# A Potent Neutralizing Nanobody Targeting the Spike Receptor-Binding Domain of SARS-CoV-2 and the Structural Basis of Its Intimate Binding

**DOI:** 10.3389/fimmu.2022.820336

**Published:** 2022-05-18

**Authors:** Jing Yang, Sheng Lin, Honglu Sun, Zimin Chen, Fanli Yang, Xi Lin, Liyan Guo, Lingling Wang, Ao Wen, Xindan Zhang, Yushan Dai, Bin He, Yu Cao, Haohao Dong, Xianbo Liu, Bo Chen, Jian Li, Qi Zhao, Guangwen Lu

**Affiliations:** ^1^West China Hospital Emergency Department (WCHED), State Key Laboratory of Biotherapy, West China Hospital, Sichuan University, Chengdu, China; ^2^Disaster Medicine Center, West China Hospital, Sichuan University, Chengdu, China; ^3^Laboratory of Aging Research and Cancer Drug Target, State Key Laboratory of Biotherapy and Cancer Center, National Clinical Research Center for Geriatrics, West China Hospital, Sichuan University, Chengdu, China; ^4^Antibody R&D Department, CHENGDU NB BIOLAB CO., LTD, Chengdu, China; ^5^School of Basic Medical Sciences, Chengdu University, Chengdu, China; ^6^College of Food and Biological Engineering, Chengdu University, Chengdu, China

**Keywords:** SARS-CoV-2, spike receptor-binding domain, nanobody, neutralization, structural basis

## Abstract

The continuous spread of severe acute respiratory syndrome coronavirus 2 (SARS-CoV-2) around the world has raised unprecedented challenges to the human society. Antibodies and nanobodies possessing neutralization activity represent promising drug candidates. In this study, we report the identification and characterization of a potent SARS-CoV-2 neutralizing nanobody that targets the viral spike receptor-binding domain (S-RBD). The nanobody, termed as Nb-007, engages SARS-CoV-2 S-RBD with the two-digit picomolar binding affinity and shows outstanding virus entry-inhibition activity. The complex structure of Nb-007 bound to SARS-CoV-2 S-RBD reveals an epitope that is partially overlapping with the binding site for the human receptor of angiotensin-converting enzyme 2 (ACE2). The nanobody therefore exerts neutralization by competing with ACE2 for S-RBD binding, which is further ascertained by our *in-vitro* biochemical analyses. Finally, we also show that Nb-007 reserves promising, though compromised, neutralization activity against the currently-circulating Delta variant and that fusion of the nanobody with Fc dramatically increases its entry-inhibition capacity. Taken together, these data have paved the way of developing Nb-007 as a drug-reserve for potential treatment of SARS-CoV-2 related diseases.

## Introduction

The pandemic of coronavirus disease 2019 (COVID-19) caused by severe acute respiratory syndrome coronavirus 2 (SARS-CoV-2) is still surging globally, posing great challenges to public health, economic activity and social order (1–3). As of 19^th^ November 2021, this highly contagious virus has caused more than 255 million infections and claimed over 5.1 million lives (https://covid19.who.int). Through the concerted efforts worldwide, several preventive vaccines have been approved for emergency use in many countries (4, 5). The administration of vaccines indeed largely alleviates the global pressure of SARS-CoV-2 spread and infection. However, with the circulation of several SARS-CoV-2 variants, especially the emergence of the Delta variant (B.1.617.2 lineage) which shows higher transmissibility among individuals (6, 7) and some resistance to immunity elicited by vaccines (8, 9), it is now not rare cases that the breakthrough infections in the fully vaccinated are observed (10, 11). It is therefore still of essential need to develop effective therapeutics to help curb the current pandemic (12).

Entry is the initial step of infection which plays a key role in the viral life cycle (13). Similar to other coronaviruses, SARS-CoV-2 utilizes its surface-located trimeric spike (S) protein to recognize the host cell receptor/(s) and further trigger membrane fusion, subsequently establishing infection (14, 15). Previous studies have shown that the receptor recognition process is mainly mediated by the binding of spike receptor-binding domain (S-RBD) to the peptidase domain of human angiotensin-converting enzyme 2 (ACE2) (16, 17). Therefore, blocking the interaction between S-RBD and ACE2 represents a promising approach for developing therapeutic or prophylactic reagents [e.g., neutralizing monoclonal antibodies (18), recombinant ACE2 protein (19), RBD-based vaccine (20), etc.] against COVID-19.

V_H_H antibodies, or nanobodies, are the antigen binding fragment of heavy-chain-only antibodies derived from camelids and cartilaginous fish (21). Compared to traditional human or murine immunoglobulin G (IgG) antibodies, nanobodies generally have several unique advantages, e.g., smaller size (~15 kDa), higher thermostability and solubility, better tissue-penetration property and simple way of expression in bacteria, etc. (22). In addition, nanobodies could be easily engineered into multimeric forms to increase the functional efficacy or half-life (commonly would be transformed into bivalent Fc fusion protein) (23). Several nanobody-derived therapeutic agents for human diseases involving inflammation, lung diseases, oncology and infectious diseases are under clinical investigation (24). Of note, the caplacizumab, a nanobody-based drug, has already been approved for thrombotic thrombocytopenic purpura therapy in Europe and USA (24). Moreover, nanobodies which can be administered by nebulized inhalation have become a promising drug candidate against respiratory viral infections including respiratory syncytial virus, influenza virus, SARS-CoV-2, etc (25–27). Currently, nanobodies targeting S-RBD of the original strain of SARS-CoV-2 have been reported by some research groups (28–31). But, with the widespread circulation of SARS-CoV-2 Delta variant, the neutralizing efficacy and potency of these nanobodies against the Delta variant need to be further evaluated. In addition, new nanobodies that can inhibit SARS-CoV-2 infection, which could act as an important drug reserve against the novel coronavirus, need to be further identified and characterized.

In this study, we reported the identification of 10 nanobodies with unique sequences from one alpaca immunized with the SARS-CoV-2 S-RBD. One of the nanobodies, Nb-007, showed high-affinity of two-digit picomolar binding to S-RBD and potent neutralizing efficacy against pseudotyped SARS-CoV-2 virus. Structural and functional studies revealed that Nb-007 could directly abolish ACE2 engagement by targeting an epitope on S-RBD that are closed to and partly overlapping with the ACE2 binding site. In addition, we also showed that Nb-007 retained S-RBD binding and neutralizing activity towards the Delta variant of SARS-CoV-2, and that its neutralizing efficacy could be significantly improved by bivalency *via* Fc-fusion.

## Materials and Methods

### Alpaca Immunization and Phage Display Screen

The SARS-CoV-2 S-RBD specific nanobody repertoire was obtained from CHENGDU NB BIOLAB CO., LTD *via* immunizing an alpaca with recombinant SARS-CoV-2 S-RBD protein. In brief, alpaca was immunized subcutaneously with 0.5 mg S-RBD protein in the presence of CFA at day 0, and then, boost immunized with 0.25 mg S-RBD protein in the presence of IFA on day 21 and 42. On day 7 after the final boost shot, ~50 ml blood from the animal was collected for the isolation of peripheral blood mononuclear cells (PBMCs). The RNA was purified from the mononuclear cells using RNAiso Plus (Takara) and was reverse-transcribed into cDNA using the PrimeScript™ II 1st Strand cDNA Synthesis Kit (Takara). The VHH genes were PCR amplified and then constructed into pComb3XSS vector. The ligated vectors were electroporated into TG1 competent cells to prepare the phage libraries. After two rounds of selection, individual phage clones were rescued and tested for the initial binding to S-RBD.

### Gene Cloning, Expression and Protein Purification

10 nanobodies (named as Nb-001 to Nb-010) were randomly selected from the unique-sequence nanobody repertoire. For recombinant nanobody preparations, the individual coding fragments with a sequence for a C-terminal 6×His tag (the residual His tag on nanobody is retained in all the subsequent experiments) were cloned into pNCMO2 vector and further introduced into *Brevibacillus choshinensis* SP3 cells for protein expression as previously reported (32). Subsequently, nanobody proteins were purified from cell culture supernatants by Ni-TED NUPharose FF beads (NUPTEC) before injection onto a Superdex™ 75 10/300 GL column (GE Healthcare) in 10 mM HEPES-NaOH (pH 7.5), 150 mM NaCl buffer.

We also prepared several mutant Nb-007 proteins (Nb-007/I26D, Nb-007/I26S, Nb-007/S27D, Nb-007/R97D and Nb-007/R97E) and the protein of a previously well-characterized S-RBD targeting nanobody Nb20 (28) in this study. The individual coding fragments with a sequence for the C-terminal 6×His tag were cloned into the pET-21a vector and subsequently transformed into *Escherichia coli* BL21 (DE3). The protein expression was conducted with the addition of 400 μM isopropyl-β-D-thiogalactoside (IPTG) followed by induction at 18°C (for Nb-007 mutants) or 37°C (for Nb20) overnight. For Nb-007 mutants, cells were harvested, lysed by sonication in re-suspension buffer composed of 10 mM HEPES-NaOH (pH 7.5) and 300 mM NaCl, and clarifed *via* centrifugation at 16 000 rpm for 40 minutes. Proteins were then purified by Ni-TED NUPharose FF beads before injection onto a Superdex™ 75 10/300 GL column in 10 mM HEPES-NaOH (pH 7.5), 150 mM NaCl buffer. For Nb20, the nanobody was expressed as inclusion body, which was extracted and refolded as previously described (33). In brief, aliquots of inclusion body were diluted dropwise into a stirring refolding buffer [100 mM Tris-HCl (pH 8.0), 400 mM L-Arg HCl, 2 mM EDTA, 5 mM reduced glutathione, 0.5 mM oxidized glutathione] and incubated overnight. Subsequently, the refolded protein was concentrated using a 5-kDa cutoff membrane with an Amicon Stirred Cell concentrator (Merck Millipore) and then exchanged into a buffer consisting of 10 mM HEPES-NaOH (pH 7.5), 150 mM NaCl. The protein was then further purified by gel filtration with a Superdex™ 75 10/300 GL column.

SARS-CoV-2 original strain S-RBD (residues 320-537 in spike protein, GenBank accession number: MN908947.3), Beta variant (B.1.351 lineage) S-RBD (residues 320-545 in spike protein and bearing K417N, E484K and N501Y mutations), Delta variant (B.1.617.2 lineage) S-RBD (residues 320-537 and harboring L452R and T478K mutations) and human ACE2 peptidase domain (PD) (residues 19-615, GenBank accession number: BAB40370.1) proteins used for further experiments were expressed in *Spodoptera frugiperda* Sf9 cells using the Bac-to-Bac baculovirus expression system (Invitrogen). The coding sequences for S-RBD which were fused in a tandem manner with an N-terminal GP67 signal peptide, a Trx tag, a 6×His tag and an Enterokinase (EK) cleavage cite were inserted into pFastBac1 vector to facilitate protein secretion, folding, purification and tag removal. The coding gene of ACE2 was inserted into pFastBac1 vector with an N-terminal GP67 signal peptide to facilitate protein secretion and a C-terminal 6×His tag for protein purification. Transfection, virus amplification and recombinant protein production were conducted with Sf9 cells. Cell culture supernatants were collected at 72 hours after infection and injected into a 5-ml His-Trap excel column (GE Healthcare) for initial purification. Subsequently, S-RBD proteins were treated by EK protease (a gift from the laboratory of Li Yang, Sichuan University) overnight at 18°C to remove Trx and His tags, and further purified on a Superdex 200 Increase 10/300 GL column (GE Healthcare) with buffer containing 10 mM HEPES-NaOH (pH 7.5) and 150 mM NaCl. For ACE2, the protein purified from His-Trap was loaded on a Source 15Q column (GE Healthcare) for ion-exchange chromatography and then injected into a Superdex 200 Increase 10/300 GL column with the final buffer consisting of 10 mM HEPES-NaOH (pH 7.5) and 150 mM NaCl.

The plasmids for Fc-fused Nb-007 and Fc-fused ACE2 were individually constructed by inserting the coding sequences into pCAGGS vector with an IL2 signal peptide at N terminus and a human IgG1 Fc fragment at C terminus. Then, the recombinant plasmids were transiently transfected into 293T cells using Lipo8000 (Beyotime). After 3 days post-transfection, the fusion proteins were purified from filtered cell culture supernatants *via* affinity chromatography using HiTrap rProtein A FF column (GE Healthcare).

### ELISA Assay

The 96-well microtiter plates (Corning) were coated with SARS-CoV-2 original strain S-RBD (200 ng per well) in 0.05 M carbonate-bicarbonate coating buffer (pH 9.6) overnight at 4℃. The wells were then blocked for 1 hour at room temperature with PBST containing 5% non-fat powdered milk (Sangon Biotech). For the binding between S-RBD and nanobodies, the indicated proteins (ACE2, Nb-001 to Nb-010) at single concentration (2 μg/ml, 100 μl per well) or 3-fold serially-diluted concentrations were added to the plates and incubated for 1.5 hours, followed by the addition of HRP-conjugated anti-His antibody (Proteintech) and incubation for another 1 hour. For the competitive binding experiment, Fc-fused ACE2 protein (1 μg/ml, 100 μl per well) were incubated in each well for 1.5 hours at room temperature. Then, 3-fold serially-diluted concentrations of Nb-007 or GST were added and incubated for 1.5 hours, followed by the addition of goat anti-human IgG-HRP (Merck Millipore) for another 1 hour. In each step, the plates were fully washed with PBST. Subsequently, TMB solution (Beyotime) was added to react with the HRP conjugates in dark at room temperature for about 3 minutes and the reaction was stopped with 2 M HCl. The emission OD450 was monitored using a microplate reader (BioTek).

### Pseudovirus Neutralization Assay

Pseudotyped SARS-CoV-2 virus [original strain, Beta variant (B.1.351 lineage) and Delta variant (B.1.617.2 lineage)] were purchased from Genomeditech (#GM-0220PV07, #GM-0220PV32, and #GM-0220PV45, respectively). The neutralization assays were performed as previously describe (19), with the HEK-293T cells that could stably express human ACE2 (HEK293T-ACE2). For pseudovirus infection, HEK293T-ACE2 cells were seeded into 96-well cell-culture plates (Corning) with 1×10^4^ cells/well and cultured at 37°C. Three-fold serially-diluted monovalent Nb-007, Nb20 or Fc-fused Nb-007 proteins were incubated with pseudovirus particles at 37°C for 60 minutes followed by adding into HEK293T-ACE2 cells. Then, the protein/pseudovirus mixtures were replaced with fresh medium after 24 hours post-infection and the cells were further cultured for another 48 hours. Luciferase activity was determined *via* the One-Lumi™ II Firefly Luciferase Assay Kit following the manufacturer’s instructions (Beyotime). The half maximal inhibitory concentration (IC_50_) values were calculated using GraphPad Prism 6.

### Syncytium Formation Assay and the Fusion Inhibition by Nb-007

The syncytium-formation inhibition activities of nanobodies (Nb-001 to Nb-010) were assessed by S-mediated cell-cell fusion assay. In brief, HEK-293T cells were co-transfected with SARS-CoV-2 original strain S and EGFP expression plasmids (HEK293T-S/EGFP cells) using Lipo8000. After 40 hours post-transfection, the HEK293T-S/EGFP cells were seeded into 96-well cell-culture plates with 2×10^4^ cells/well followed by incubation with indicated proteins (ACE2 or nanobodies) at 37°C for 1 hour. Then, the HEK293T-ACE2 cells were plated into the plates with 4×10^4^ cells/well followed by co-culturing at 37°C for another 3 hours. The final concentration of proteins were used at 1 μM or 10 μM (for initial screenings) or with 3-fold serially dilutions starting from 11.45 μM (for quantitative determination). Finally, the formation of syncytia were visualized by fluorescence microscope (Olympus). The GFP areas were quantified on ImageJ and the IC_50_ values were calculated using GraphPad Prism 6.

### Surface Plasmon Resonance (SPR) Assay

All the SPR experiments were carried out with the BIAcore 8K system (GE Healthcare). For affinity determination, S-RBDs (original strain, Beta variant and Delta variant) were individually immobilized onto the CM5 sensor chip (GE Healthcare) using the Amine Coupling Kit (GE Healthcare). Gradient concentrations of analyte [Nb-007, Nb-007 mutant (Nb-007/I26D, Nb-007/I26S, Nb-007/S27D, Nb-007/R97D or Nb-007/R97E), Nb-007-Fc or ACE2] were flowed over S-RBD in the running buffer containing 10 mM HEPES-NaOH (pH 7.5), 150 mM NaCl and 0.05% Tween-20 at a rate of 30 μl/min. After each cycle, the chip was re-generated using pH 1.5 glycine. The obtained kinetic data were further analyzed with the Biacore Insight Evaluation Software (GE Healthcare) for dissociation constant (*K*_D_) calculations, using the 1:1 (Langmuir) binding model for the slow-on/slow-off data and the steady-state affinity model for the fast-on/fast-off data, respectively. For the competitive binding experiments, SARS-CoV-2 original strain S-RBD was firstly immobilized onto CM5 sensor chip as described above. Then, the nanobody [1 μM for Nb-005 (as a non-ACE2 competing control) or 0.5 μM for Nb-007] or running buffer was individually flowed over the chip at a rate of 30 μl/min for 150 s followed by the immediate injection of ACE2 protein (at the concentration of 0.5 μM) at the same rate for another 150 s.

### Crystallization

To prepare the Nb-007/S-RBD complex for crystallization assay, SARS-CoV-2 original strain S-RBD was incubated with Nb-007 at a molar ratio of 1:1.3 at 4°C for 2 hours. Then, the mixture was subjected to the gel filtration chromatography using a Superdex 200 Increase 10/300 GL column in 20 mM Tris-HCl (pH 8.0), 150 mM NaCl buffer to further purify the complex. Fractions containing the complex were pooled and concentrated to 10 mg/ml for crystallization screenings. The initial crystallization screenings were conducted using the commercial crystallization kits (Hampton Research and Molecular Dimensions) by the vapour-diffusion sitting-drop method. In brief, 1 μl Nb-007/S-RBD complex was mixed with 1 μl reservoir solution, and the subsequent mixture was then equilibrated against 70 μl reservoir solution at 18°C. The diffractable crystals for the complex were grown in the condition consisting of 0.2 M NaCl (pH 6.9) and 20% w/v PEG 3350.

### Data Collection and Structure Determination

For data collection, the crystals were briefly soaked in reservoir solution supplemented with 20% (v/v) glycerol and then flash-cooled in liquid nitrogen. X-ray diffraction data were collected at Shanghai Synchrotron Radiation Facility (SSRF) beamline BL18U1 and processed with HKL3000 (34) for indexing, integration, and scaling. The structure of Nb-007/S-RBD complex was determined by molecular replacement with the Phaser program (35) from the CCP4 suite (36), using the previously reported SARS-CoV-2 RBD structure (PDB code: 6YZ5) (30) and nanobody structure (PDB code: 6B20) (37) as the search model. The atomic model was further completed with Coot (38) and refined with phenix.refine in Phenix (39). The final data processing and structure refinement statistics are summarized in **Supplementary Table 1**. All structural figures were generated using PyMOL (https://pymol.org/).

## Results

### Identification of a Neutralizing Nanobody Targeting SARS-CoV-2 S-RBD

**Figure 1:**
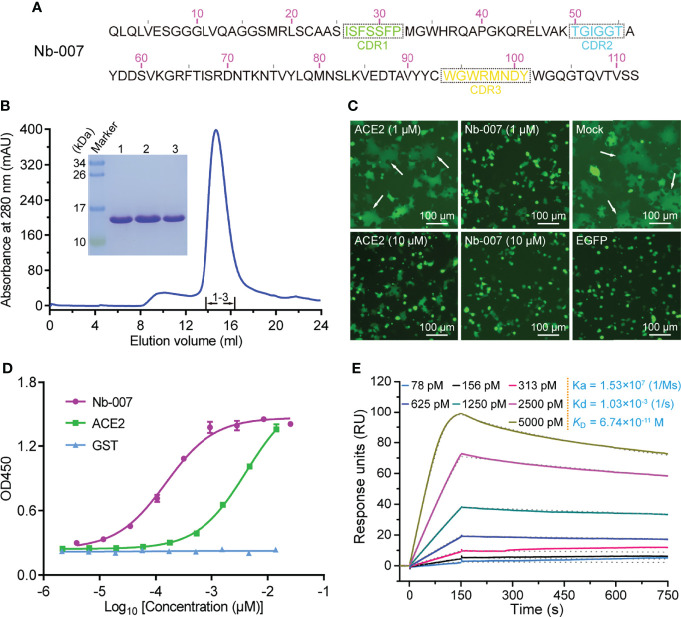
Identification and functional characterization of Nb-007 as a neutralizing nanobody with high binding affinity towards SARS-CoV-2 S-RBD. **(A)** The amino-acid sequence of Nb-007. The three complementarity-determining regions (CDRs) are labelled with dashed boxes. **(B)** Characterization of the solution behavior of Nb-007 by gel filtration chromatography. The inset figure shows the SDS-PAGE analyses of the pooled samples. **(C)** Inhibition of the SARS-CoV-2 S-protein-mediated syncytium-formation in the presence of Nb-007 or ACE2 at the indicated concentrations. Mock: cell-cell fusion induced by mixing HEK293T-ACE2 and HEK293T-S/EGFP cells without the addition of either the nanobody or ACE2. EGFP: only 293T-S/EGFP cells. The representative syncytia are marked with white arrows. Scale bar equals 100 µm. **(D)** The multi-concentration ELISA-binding profile between SARS-CoV-2 S-RBD and the indicated proteins (Nb-007, ACE2 and GST). The OD450 emissions are plotted as curves. Each error bar represents the mean ± SD from three independent experiences. **(E)** Interaction between Nb-007 and SARS-CoV-2 S-RBD detected *via* SPR. The recorded binding profiles and calculated kinetic parameters are shown.

In order to produce the SARS-CoV-2 S-RBD-specific nanobodies, we immunized an alpaca with recombinant RBD protein and constructed a phage display V_H_H library followed by two consecutive rounds of bio-panning using ELISA-based screen. Then, we selected 10 nanobodies (named as Nb-001 to Nb-010) randomly from unique-sequence nanobody repertoire (**Figure 1A**; **Supplementary Figure 1**), and prepared them to high-purity from *brevibacillus* cells for further functional verification (**Figure 1B**; **Supplementary Figure 2**).

The specific binding of the prepared nanobodies to SARS-CoV-2 S-RBD was first verified by a single-concentration ELISA-binding assay. As expected, most of obtained nanobodies, except Nb-009, could interact with S-RBD (**Supplementary Figure 3**). Subsequently, we performed the S-mediated cell-cell fusion assay for the initial screening of nanobodies with potential neutralizing activities. Because the ectodomain protein of ACE2 could function as a decoy by competing for S-RBD binding with the cell surface ACE2 receptor (19, 40), we therefore selected the recombinant ACE2 protein as a positive control in the assay. As shown in **Figure 1C**, the S-mediated syncytium formation was observed to be largely inhibited in the presence of 10 μM ACE2 but only marginally affected by 1 μM ACE2. In contrast, Nb-007 was shown to completely block the formation of syncytium at 1 μM concentration, demonstrating that the nanobody possessed more superior inhibitory activity than recombinant ACE2. However, no obvious inhibition of the syncytium formation was observed in other groups even at the 10-μM nanobody concentration (**Supplementary Figure 4**), indicating that these nanobodies target some non-neutralizing epitopes on S-RBD.

Subsequently, the binding characteristics between Nb-007 and S-RBD was further characterized *via* the multi-concentration ELISA and the surface plasmon resonance (SPR) assays. The ELISA result revealed that, comparing to ACE2, Nb-007 could recognize and engage S-RBD with much better efficiency (**Figure 1D**). The real-time SPR data further showed that the equilibrium dissociation constant (*K*_D_) between Nb-007 and S-RBD was 67.4 pM (**Figure 1E**). This value represents an affinity that is much higher (about three orders of magnitude) than that between ACE2 and S-RBD (**Supplementary Figure 5**). Collectively, these results indicate that Nb-007 is a potent neutralizer for SARS-CoV-2.

### Potent Neutralizing Efficacy of Nb-007 Against SARS-CoV-2

**Figure 2:**
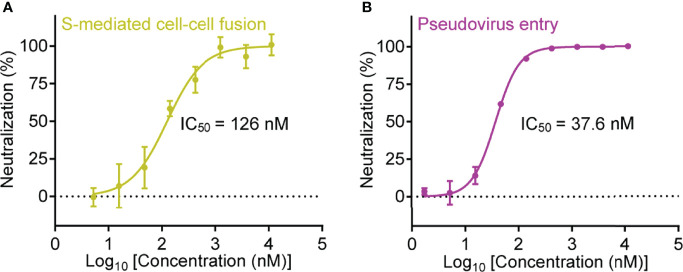
Quantitative analysis of the inhibitory activity of Nb-007. **(A)** Inhibition of the SARS-CoV-2 S-protein-mediated cell-cell fusion with 3-fold serial dilutions of Nb-007 at the indicated concentrations. Error bar stands for the mean ± SD from triplicate experiments. **(B)** Inhibition of the SARS-CoV-2 pseudovirus entry with 3-fold gradient dilutions of Nb-007 at the indicated concentrations. Error bar shows the mean ± SD of triplicate.

Inspired by the high binding affinity between Nb-007 and SARS-CoV-2 S-RBD, we further quantitatively investigated the functional inhibitory activity of Nb-007 by the cell-cell fusion and pseudovirus-entry assays. As expected, both the S-mediated fusion and the S-facilitated entry could be effectively inhibited by the nanobody. The half maximal inhibitory concentration (IC_50_) was determined to be 126 nM in our cell-cell fusion inhibition assay and 37.6 nM in the pseudovirus infection-inhibition assay (**Figure 2**), demonstrating that Nb-007 is indeed a neutralizing nanobody against SARS-CoV-2.

It is notable that the IC_50_ value evaluated *via* the pseudovirus neutralization assay might vary due to the differences in the experimental-conditions and/or the materials used (e.g., the time for infection, the type or dosage of pseudovirus, the type, number or state of the targeted cell, etc.). For instance, the “neutralizing potency” reported by different groups for the same decoy ACE2-Fc as a neutralizer against SARS-CoV-2 pseudovirus indeed varied a lot (41, 42). We therefore prepared a previously-reported highly-potent nanobody named Nb20 (also an S-RBD targeting nanobody) (28) and determined its neutralizing activity *via* our pseudovirus-entry-inhibition assay. Unexpectedly, the final IC_50_ for Nb20 was calculated to be 3.08 nM (**Supplementary Figure 6**), which was about 30-fold lower than that reported in the previous publication (an IC_50_ of about 0.102 nM) (28). The results highlighted that Nb-007, with an affinity of 67.4 pM to S-RBD and an IC_50_ of 37.6 nM determined *via* our pseudovirus system, indeed showed high neutralization potency.

### Complex Structure of Nb-007 Bound to SARS-CoV-2 S-RBD

**Figure 3:**
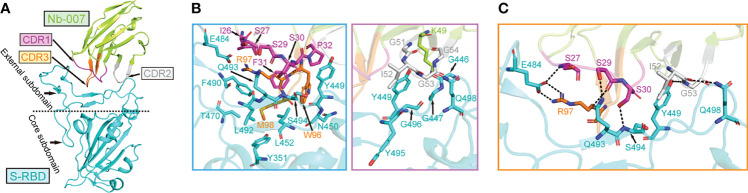
The complex structure of Nb-007 bound to SARS-CoV-2 S-RBD. **(A)** A cartoon representation of the complex structure. Nb-007 is colored in lemon and S-RBD in cyan. The CDR1, CDR2 and CDR3 of Nb-007 are indicated with arrows and highlighted in magenta, gray and orange, respectively. The external subdomain and core subdomain of S-RBD, are roughly divided by a dashed line. **(B)** The multiple van der Waals (vdw) and hydrophobic interactions between Nb-007 and S-RBD. The left panel, amino acid interactions between CDR1 and CDR3 of Nb-007 and S-RBD. The right panel, amino acid interactions between framework region and CDR2 of Nb-007 and S-RBD. Those residues providing ≥2 contacts are shown (the distance cutoff is 4.5 Å). **(C)** Hydrophilic interactions (hydrogen bonds and salt bridges) between Nb-007 and S-RBD (the distance cutoff is 3.1 Å).

In order to learn the interactive binding mode between Nb-007 and S-RBD in detail, we determined the crystal structure of Nb-007/S-RBD complex at 2.0-Å resolution. The structure was solved by molecular replacement, and the final model was refined to *R*_work_ = 0.199 and *R*_free_ = 0.223, respectively (**Supplementary Table 1**). The complex structure contains one Nb-007 nanobody bound to a single S-RBD molecule in the asymmetric unit (**Figure 3A**). The electron densities for amino acids ranging from L2 to S112 in the Nb-007 chain and residues spanning N334 to P527 in the S-RBD molecule could be clearly traced. The previously reported N-linked glycans at the N343 residue of S-RBD is nonetheless density-untraceable in our final model.

In the solved structure, S-RBD represents as two-structural-entity assemblies, which are composed of a conserved core subdomain and a canonical external subdomain. The Nb-007 nanobody utilizes its three CDRs to interact with S-RBD, obliquely inserting the CDR loops into the large concave depression in the external subdomain of S-RBD (**Figure 3A**). The paratope on Nb-007 is composed of 14 amino acids, forming multiple van der Waals (vdw) and hydrophobic contacts with S-RBD. These numerous engagements involve residues of Nb-007: I26-S27 and S29-P32 in CDR1, K49 in the nanobody framework-region, G51-G54 in CDR2, and W96-M98 in CDR3, interacting with amino acids of S-RBD: Y351 in the core subdomain and G446-G447, Y449-N450, L452, T470, E484, F490, L492-G496 and Q498 in the external subdomain (**Figure 3B**). In addition, a total of eight strong hydrophilic interactions (hydrogen bonds and salt bridges), which are mediated by Nb-007-S27 with S-RBD-E484, S29 with Q493, S30 with S494, I52 with Q498, G53 with Y449, and R97 with E484, respectively, were observed to further stabilize the nanobody/S-RBD engagement (**Figure 3C**). Taken together, these intimate inter-molecule contacts upon complex formation well explain the superior S-RBD binding capacity of Nb-007 observed in our *in-vitro* binding assays.

### Molecular Basis of SARS-CoV-2 Neutralization by Nb-007

**Figure 4:**
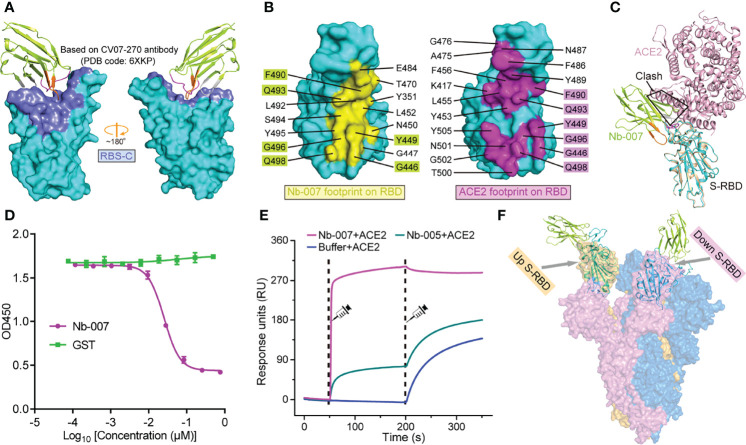
Molecular basis for Nb-007 neutralization. **(A)** The previously identified RBS-C epitope [Based on CV07-270 antibody (PDB code: 6XKP)] is colored by slate and mapped onto our Nb-007/S-RBD complex. The relative orientation of RBD is the same as in **Supplementary Figure 7**. Nb-007 is shown in cartoon representation and colored as in **Figure 3**. **(B)** The footprints of Nb-007 (left panel, highlighted in yellow) and ACE2 (right panel, highlighted in magenta) on S-RBD. The involved residues are marked and the overlapping amino acids are highlighted. **(C)** Superimposition of the complex structures of Nb-007/S-RBD and ACE2/S-RBD. Steric clashes between Nb-007 and ACE2 are highlighted. **(D)** Competitive binding assays by ELISA. SARS-CoV-2 S-RBD was coated on 96-well plates, recombinant Fc-fused ACE2 was first added, followed by serial dilutions of Nb-007. Error bar stands for the mean ± SD. Experiments were performed in triplicate. **(E)** SPR kinetics of competitive binding of Nb-007 and ACE2 to SARS-CoV-2 S-RBD. S-RBD was immobilized onto a sensor chip. The indicated nanobodies (Nb-007 and Nb-005) and ACE2 were successively injected. The real-time binding profiles are recorded. Clearly shown is that Nb-007 but not Nb-005 interferes with ACE2 binding. **(F)** Alignment of the Nb-007/S-RBD structure (shown in cartoon) onto a previously solved cryo-EM structure of the SARS-CoV-2 S-trimer (shown in surface, PDB code: 6VYB). The up- and down-conformation of the S-RBD are highlighted.

Based on the binding interface and the footprint position of known antibodies on SARS-CoV-2 S-RBD, the antigenic sites of S-RBD could be clustered into five regions, which were previously termed as RBS-A, RBS-B, RBS-C, CR3022 site and S309 site, respectively (43, 44) (**Figure 4A**; **Supplementary Figure 7**). Facilitated by the solved structure, we further compared the binding site of Nb-007 on S-RBD with these identified epitopes (exemplified by the interface of a representative antibody from each group on S-RBD). We found that the epitope of S-RBD that Nb-007 binds to mostly matched the RBS-C interface and should therefore be assigned to this group (**Figure 4A**). Meanwhile, in parallel comparison between Nb-007 and reported representative antibodies (including CB6, CV07-250, CV07-270, P2B-2F6, CR3022, S309 and A23-58.1) or nanobodies (including Ty1, Nb20, Nb12, Nb30, VHHE, Re5D06 and Re9F06) revealed that, for both the recognized epitopes on S-RBD and the bound angle of the antibody/nanobody, Nb-007 resembled and mostly mimicked the CV07-270 and P2B-2F6 antibodies as well as the Ty1 nanobody (**Supplementary Figure 8**).

According to previous studies, the footprints of antibodies in the RBS-C group on S-RBD are normally partially-overlapping with the ACE2 binding site, thereby exerting neutralization by competing against the RBD/ACE2 engagement (43, 44). In addition, antibodies CV07-270, P2B-2F6 and nanobody Ty1 all possess an overlapped binding interface with that of ACE2 and subsequently compete against receptor binding for neutralization (18, 31, 45). These observations remind us of a potential competition between Nb-007 and ACE2 for S-RBD binding to neutralize virus infection. We therefore mapped the binding sites of Nb-007 and ACE2 on S-RBD for comparison. Of the 15-residue footprint for Nb-007 and the 19-amino-acid footprint for ACE2, the interfaces covering residues G446, Y449, F490, Q493, G496, and Q498 are overlapping (**Figure 4B**). In addition, superimposition of the two complex structures between Nb-007/S-RBD and ACE2/S-RBD clearly showed that steric hindrance would occur between Nb-007 and ACE2 (**Figure 4C**). These structural analyses indicate that the Nb-007 nanobody should exert its neutralizing activity by competing with ACE2. Consistent with structural observations, our competitive binding assays, including ELISA and SPR both verified that Nb-007 could efficiently block the ACE2/S-RBD interaction (**Figures 4D, E****)**. Noted that RBD on the spike trimer has two states, including an ACE2 inaccessible, down conformation and an ACE2 accessible, up conformation, we further aligned our Nb-007/S-RBD structure to a previously reported cryo-electron microscopy structure of SARS-CoV-2 S-trimer (PDB code: 6VYB) (46), in which one RBD was in the up state and the other two were in the down state (**Figure 4F**). The structural superimposition revealed that, unlike ACE2, Nb-007 could bind S-RBD of both up and down conformations without obvious steric hindrance with other spike protomers. In summary, our structural and functional data clearly showed that Nb-007 could directly compete with ACE2 for spike/S-RBD engagement.

### Neutralizing Ability of Nb-007 Against Circulating SARS-CoV-2 Variants

**Figure 5:**
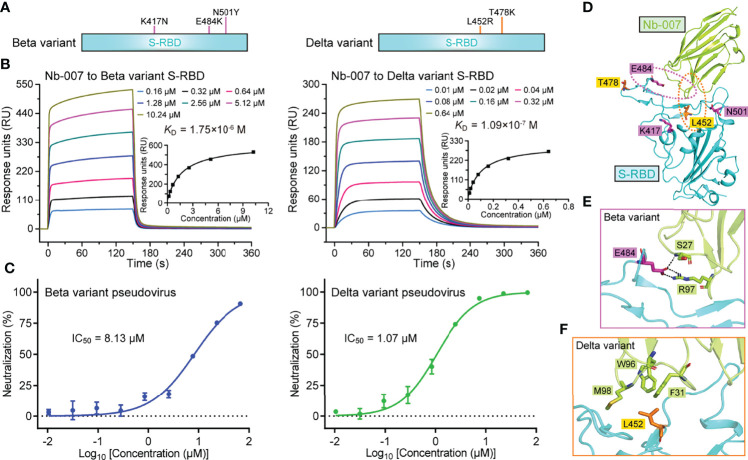
Binding capacity and neutralizing activity of Nb-007 against SARS-CoV-2 variants. **(A)** Schematic view of the SARS-CoV-2 variants highlighting the mutations identified in S-RBD. Left panel: Beta variant. Right panel: Delta variant. **(B)** Affinity analysis of the binding of Nb-007 to the indicated SARS-CoV-2 variant S-RBD using SPR. The real-time binding kinetics are shown. **(C)** Inhibition of the pseudovirus entry by Nb-007 at the indicated concentrations for the SARS-CoV-2 variants. Each error bar represents the mean ± SD of triplicate. **(D)** An overview of the steric positions for those mutations identified in the SARS-CoV-2 variants relative to the bound nanobody. The three mutations in the Beta variant and two mutations in the Delta variant are shown as sticks and colored magenta and orange, respectively. Residues E484 and L452 are highlighted and their amino acid interaction details with the bound nanobody are further presented in **(E, F)**, respectively. **(E)** The interactions between S-RBD E484 and Nb-007 S27 and R97. **(F)** The interactions between S-RBD L452 and Nb-007 F31, W96, and M98.

Currently, several natural SARS-CoV-2 variants carrying the S-RBD mutations have emerged along with the persistent spread of the virus. Among the multiple circulating variants, Beta (B.1.351 lineage) and Delta (B.1.617.2 lineage) variants are of particular concern because of their increased ability of immune escape and the enhanced infectivity (7, 47). In S-RBD, the Beta variant bears K417N/E484K/N501Y mutations and the Delta variant harbors L452R/T478K mutations (**Figure 5A**). It would be of particular interest to investigate whether Nb-007 still reserves binding capacity to the mutant S-RBDs. The binding affinities of Nb-007 engaging S-RBD of the Beta and Delta variants were subsequently determined by SPR. Although Nb-007 could engage both S-RBD mutants, the binding capacity was indeed affected because of the mutations. The *K*_D_ values were calculated to be 1.75 μM for the Beta variant S-RBD and 109 nM for the Delta variant S-RBD, respectively (**Figure 5B**). Echoing the decreased binding affinities, Nb-007 neutralized the pseudotyped variant viruses with reduced efficacy, showing an IC_50_ of 8.13 μM for the Beta variant and 1.07 μM for the Delta variant, respectively (**Figure 5C**).

In light of the reduced neutralizing efficacy of Nb-007 against these two circulating variants, we subsequently mapped the five S-RBD mutations (three in the Beta variant and two in the Delta variant) onto our Nb-007/S-RBD complex structure, aiming to delineate the basis for the decreased binding (**Figure 5D**). It was found that residues K417, T478, and N501 were sterically far away from Nb-007. The mutations of K417N and N501Y in the Beta variant and T478K in the Delta variant should therefore not interfere with the nanobody binding to S-RBD. In contrast, amino acids L452 and E484 both located in close proximity to Nb-007. For the Beta variant (**Figure 5E**), the E484K mutation would probably interrupt the hydrogen bond initially formed between S-RBD E484 and Nb-007 S27. In addition, substitution of the negatively-charged E484 residue with a positively-charged arginine amino acid could lead to charge repulsion towards residue R97 of Nb-007, further compromising the nanobody/S-RBD interactions. For the Delta variant (**Figure 5F**), replacement of the apolar leucine residue with a charged arginine amino acid would likely disrupt the hydrophobic interactions initially observed between L452 of S-RBD and F31, W96, and M98 of Nb-007. Moreover, the bulky side-chain of arginine might also generate some steric hindrance with the bound nanobody, thus further decreasing the binding affinity.

Noted that Nb-007 retained relatively good binding capacity (though compromised) towards Delta S-RBD but showed significantly lower binding affinity when engaging Beta S-RBD, we further targeted the S-RBD binding interface of the nanobody and designed several single-point mutants, aiming to improve its interaction with S-RBD of the Beta variant. To this end, we have successfully prepared the single-point-mutation-version of the nanobody proteins, including Nb-007/I26D, Nb-007/I26S, Nb-007/S27D, Nb-007/R97D and Nb-007/R97E. In comparison to wild-type Nb-007, however, none of the nanobody mutants showed obvious affinity-enhancement towards Beta variant S-RBD. Nevertheless, a slight improvement was observed for Nb-007/I26S (**Supplementary Figure 9**).

### Efficacy Improvement *via* Bivalency With Fc

**Figure 6:**
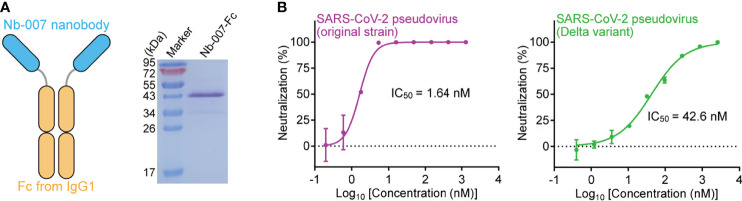
Improvement of the neutralizing activity of Nb-007 by fusion with Fc. **(A)** Diagram (left panel) showing the bivalency of Nb-007 by fusion with the Fc domain of human IgG1. The SDS-PAGE analysis of the purified Nb-007-Fc protein is shown in the right panel. **(B)** Neutralization of SARS-CoV-2 pseudovirus by Nb-007-Fc. Left panel: Nb-007-Fc against the pseudovirus of the original SARS-CoV-2 strain. Right panel: Nb-007-Fc against pseudovirus of the Delta variant. Each error bar shows the mean ± SD. Experiments were performed in triplicate.

We finally resorted to Nb-007 multimerization to improve its binding capability for S-RBD, especially those from the Beta and Delta variants. The conventional approach for increasing the nanobody binding to an antigen was to fuse the monovalent nanobody protein to the Fc domain of human IgG, thus transforming the protein into the homo-dimeric form for bivalent binding (30, 31). We therefore further fused Nb-007 with IgG1 Fc and subsequently prepared the Fc-fusion protein (Nb-007-Fc), aiming to enhance the binding affinity and neutralizing efficacy of the nanobody (**Figure 6A**). As expected, the real-time SPR data showed a remarkable enhancement in *in-vitro* binding affinity between the bivalent version of Nb-007 and the Beta or Delta variant S-RBD, with the *K*_D_ values being calculated to be 44.4 nM and 0.929 nM, respectively (**Supplementary Figures 10A, B**). Consistently, Nb-007-Fc exhibited significantly increased virus neutralizing activity towards SARS-CoV-2 of the original strain, the Beta and Delta variants. Using the pseudotyped viruses, the IC_50_ values of Nb-007-Fc were calculated to be 1.64 nM against the original SARS-CoV-2 strain, 405 nM against the Beta variant and 42.6 nM against the Delta variant (**Figure 6B**; **Supplementary Figure 10C**). These values represent about 23-fold (for original strain), 20-fold (for Beta variant) and 25-fold (for Delta variant) increase in the neutralizing activity, respectively.

## Discussion

In face of the severe social and economic threat posed by the continuous COVID-19 pandemic, great efforts have been taken among scientific community to reveal the life cycle and pathogenesis of SARS-CoV-2 (48). Owing to the intensified structural and functional studies, the underlying mechanisms of SARS-CoV-2 entry, replication and assembly have been well-elucidated (16, 46, 49–54), based on which several vaccines, monoclonal antibodies, and small-molecule drugs have been quickly developed to clinical use (55–57). The constant emergence of SARS-CoV-2 variants, however, has raised new challenges to the currently approved SARS-CoV-2 drugs, especially to the antibody-based treatment (immune escape, resistance, etc). One way to tackle the problem is to identify a large number of candidate drugs of different forms with the therapeutic potential to develop anti-SARS-CoV-2 drug reserves. Nanobody could serve as an important part of the arsenals to fight against the novel coronavirus. Nanobody possesses several superior features (e.g., low immunogenicity, simple humanization property, low-cost and scalable production ability, etc.) (58), making this unique single-chain antibody form a good option for the clinical treatment of COVID-19. In the current study, we report the identification and characterization of a potent neutralizing nanobody, Nb-007, that targets SARS-CoV-2 S-RBD. The nanobody possesses high RBD-binding affinity, interferes with the S engagement of ACE2, and exhibits potent neutralizing activity. In addition, Nb-007 also shows apparent virus entry-inhibition activity against the currently circulating SARS-CoV-2 Delta variant. *Via* fusion with Fc, we are also able to dramatically improve the binding of Nb-007 to S-RBD of the SARS-CoV-2 Beta variant. We therefore believe that the Nb-007 nanobody identified in this study represents a promising drug candidate for COVID-19 treatment.

Although Nb-007 and its Fc-fusion protein could bind Beta S-RBD and neutralize virus infection against the Beta variant, the capacity is indeed largely affected when compared to those for the prototype and the Delta-variant viruses. Our structural analyses reveal that the E484K mutation could probably disrupt the hydrogen bond interaction initially formed between E484 in S-RBD and S27 in Nb-007 and further cause charge-repulsion between the substituted K484 in S-RBD and R97 in the nanobody. Nevertheless, single mutations of I26D, S27D, R97D or R97E in Nb-007 could not improve the binding of the nanobody to Beta S-RBD, reminding us that introduction of an acidic side-chain at these positions cannot restore the salt-bridge interactions with Beta K484. The substitution of I26 with a serine, however, is observed to slightly benefit the interaction. These mutagenesis data may indicate that back-mutations with small-side-chain residues at positions 26, 27 and 97 of Nb-007, either alone or in combination, might create extra space to accommodate the large K484 amino acid of Beta S-RBD and thereby improve the binding. It’s worth noting that, besides the Beta variant, some other circulating SARS-CoV-2 variants [e.g., Gamma variant (P.1 lineage), Eta variant (B.1.525 lineage), Iota (B.1.526 lineage), Mu (B.1.621 lineage), etc.] also carry the E484K mutation on S-RBD. In such circumstances, the neutralization capacity of Nb-007 towards these variant viruses should also be compromised. Though the global circulation of these variants is limited in comparison to the Delta variant, their potential threat should never be ignored. Furthermore, we also notice that the newly-emergent Omicron variant (B.1.1.529 lineage) contains an E484A mutation on S-RBD. Unlike its substitution by a lysine residue in Beta which results in the introduction of a large basic side-chain at this position and therefore leads to potential steric hindrance and charge repulsion, however, A484 in Omicron is of small side-chain and does not possess any positive charge. In this case, we tend to believe that the adverse impact of E484A mutation in Omicron would not be as significant as that of E484K substitution in Beta. Nevertheless, we find that Omicron also contains Q493R, Q498R mutations, which are also located within the Nb-007 binding interface. A combination of these mutations would, in our opinion, also compromised Nb-007 efficacy against Omicron. Further modification and/or back-mutation work on Nb-007 to make the nanobody better tolerate the mutations (especially at position E484) in S-RBD should be explored in the future.

As a common strategy towards potential clinical applications, proteinaceous macromolecule drugs are usually modified by fusion with human IgG Fc (19, 31). In most cases, such process not only increases the stability and serum half-life of a protein but also improves the efficacy because of the Fc-mediated dimerization and thereby of active-site bivalency. Accordingly, we indeed find that fusion of Nb-007 with human IgG1 Fc could dramatically increase its virus entry-inhibition activity towards SARS-CoV-2 of the original strain and Delta variant. In addition, the bivalent modification of Nb-007 also transforms the nanobody into a much better S-RBD binder and SARS-CoV-2 neutralizer against the Beta variant. As a matter of fact, several previous studies have already shown that the protein-multivalency strategy could transform SARS-CoV-2 nanobodies into ultra-potent neutralizers (28, 29). Since multiple epitopes (RBS-A, RBS-B, RBS-C, CR3022 site and S309 site) have now been characterized on S-RBD (43, 44), the combined use of Nb-007 with other non-overlapping-epitope nanobodies (especially those targeting the conserved regions on S-RBD) to form nanobody cocktails or to constitute a bi-specific or multi-specific antibody should not only enhance neutralizing efficacy but also increase the mutational barrier to the virus, which might be explored in the future.

In summary, we have reported the identification and characterization of a potent SARS-CoV-2 neutralizing nanobody targeting S-RBD, which we believe could serve as an anti-viral drug reserve for potential treatment of COVID-19.

## Data Availability Statement

The structure of this study can be found in the Protein Data Bank with 7W1S access code.

## Author Contributions

GL conceived the idea and supervised the whole project. JY, HS, and SL conducted the majority of the experiments. SL collected the datasets and solved the structure. ZC and FY facilitated pseudovirus neutralization assay. XL and LG supported syncytium-formation inhibition assay. LW, AW, XZ, and YD assisted with the protein preparation and cell maintenance. XBL and BC performed the alpaca immunization and the bio-panning experiments. GL, SL, and JY wrote the manuscript. BH, YC, HD, JL, and QZ provided the reagents and participated in experimental design as well as manuscript editing and discussion. All authors contributed to the article and approved the submitted version.

## Funding

This work was supported by the National Key Research and Development Program of China (Grant no. 2021YFC2301402), the National Natural Science Foundation of China (Grant no. 82041042), the special research fund on COVID-19 of West China Hospital, Sichuan University (Grant no. HX-2019-nCoV-004), and the 1.3.5 project for disciplines of excellence, West China Hospital, Sichuan University (Grant no. ZYYC20008).

## Conflict of Interest

Authors XBL and BC are employed by CHENGDU NB BIOLAB CO., LTD. The remaining authors declare that the research was conducted in the absence of any commercial or financial relationships that could be construed as a potential conflict of interest.

## Publisher’s Note

All claims expressed in this article are solely those of the authors and do not necessarily represent those of their affiliated organizations, or those of the publisher, the editors and the reviewers. Any product that may be evaluated in this article, or claim that may be made by its manufacturer, is not guaranteed or endorsed by the publisher.
