# Supplementary material for: A Potent Neutralizing Nanobody Targeting the Spike Receptor-Binding Domain of SARS-CoV-2 and the Structural Basis of Its Intimate Binding
Source: Front Immunol. 2022 May 18;13:820336. doi: 10.3389/fimmu.2022.820336 (PMC9158119; doi:10.3389/fimmu.2022.820336)
Supplement: Supplementary file 1 [file DataSheet_1.pdf]

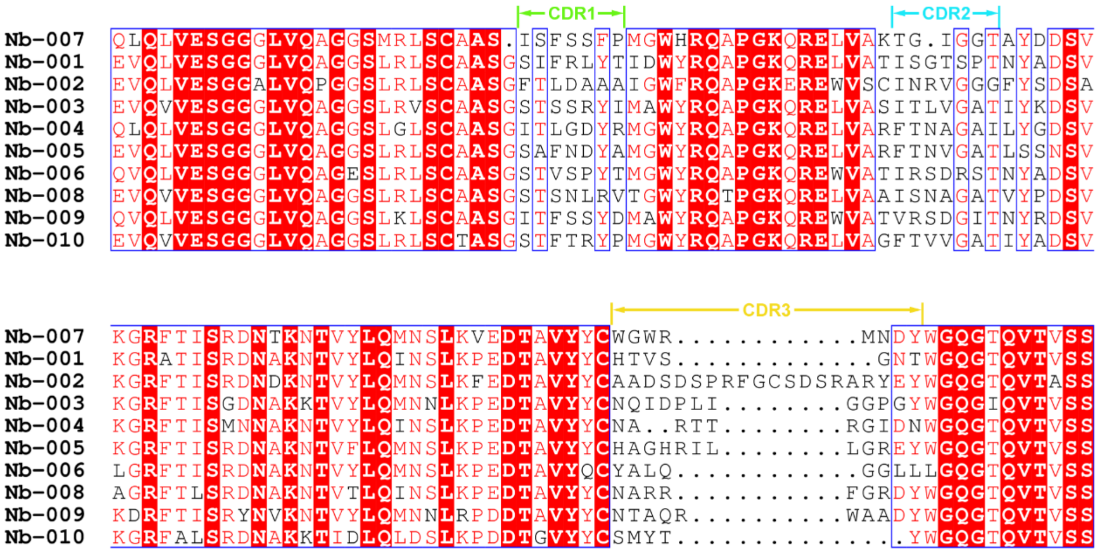

Supplementary Figure 1. Multiple sequence alignment of the ten nanobodies with unique sequences identified in this study. The CDR regions are marked.

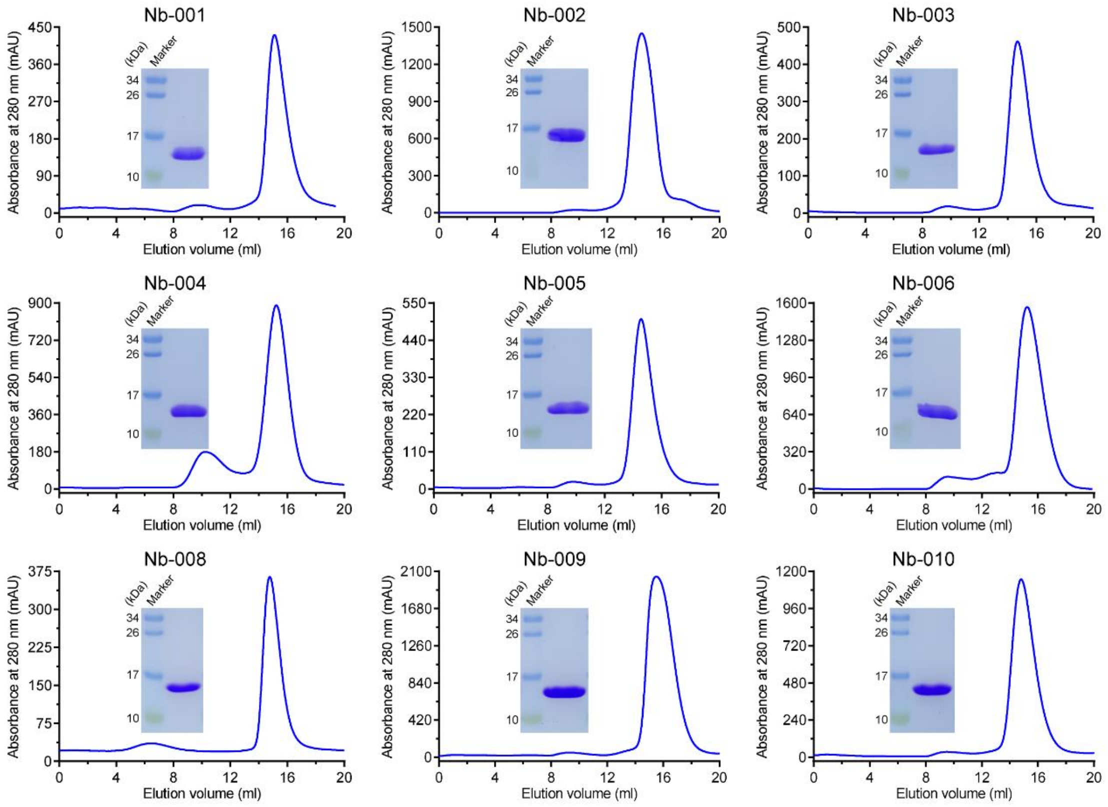

Supplementary Figure 2. Purification and gel-filtration characteristics of the indicated nanobodies. Solution behaviors of the 9 non-neutralizing nanobodies identified in the study on a Superdex^{TM} 75 10/300 GL column are shown. The inset figure shows the SDS-PAGE analyses of the pooled samples.

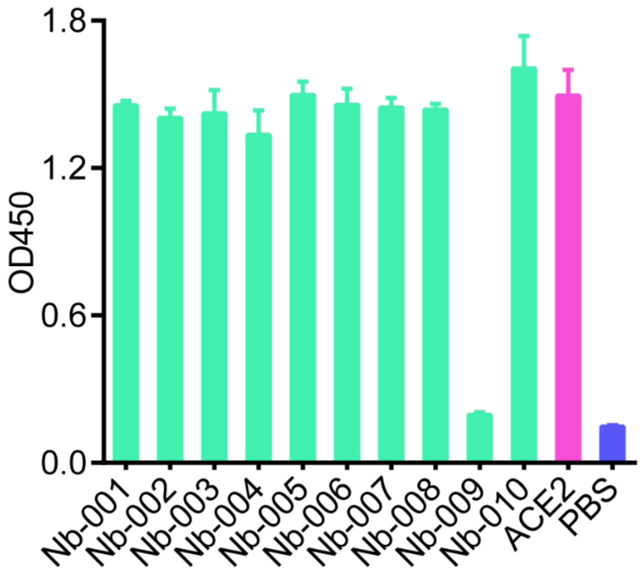

Supplementary Figure 3. Binding of the indicated nanobodies to SARS-CoV-2 S-RBD verified by single-concentration ELISA-binding assay. The experiments were performed with 200 ng SARS-CoV-2 S-RBD immobilized on the plate and 0.2 μg nanobodies (green) or ACE2 (magenta) as the analyte. The emission OD450 was plotted as histograms. The error bar shows the mean ± SD from three independent experiments.

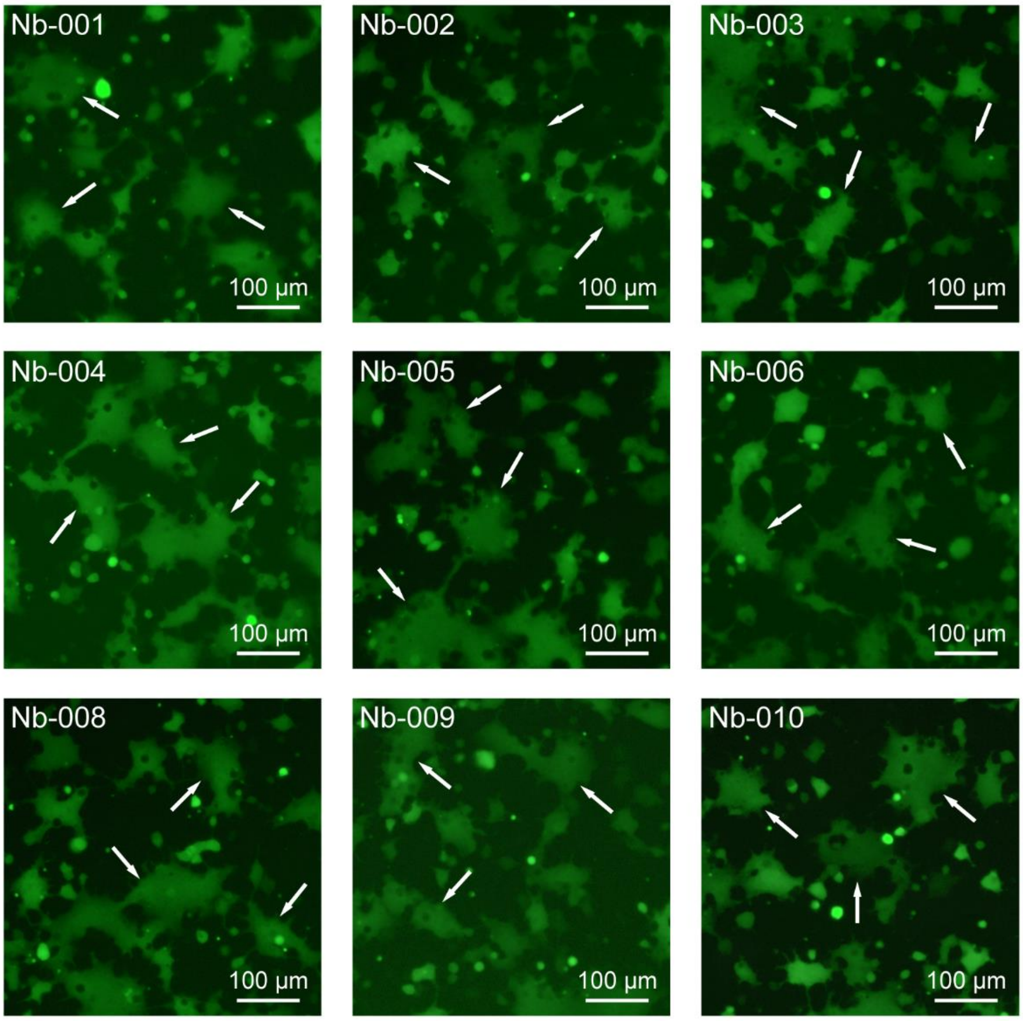

Supplementary Figure 4. Initial screenings for the potential virus-entry inhibition by the indicated nanobodies based on the S-mediated syncytium-formation assay. SARS-CoV-2 S-mediated cell–cell fusion experiments were performed in the presence of 10 μM nanobody. The formed syncytia are marked with white arrows. Scale bar equals 100 μm.

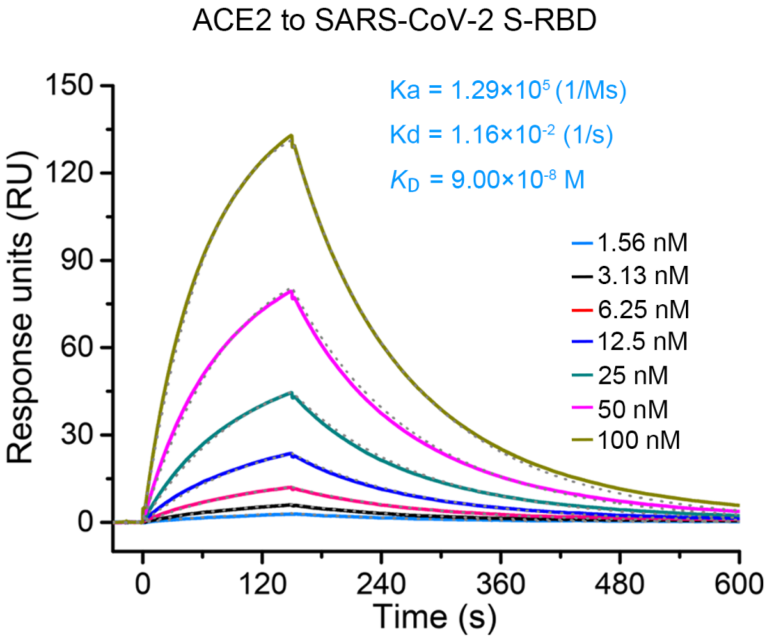

Supplementary Figure 5. Binding capacity of ACE2 to SARS-CoV-2 S-RBD detected via SPR. The recorded binding profiles and calculated kinetic parameters are shown.

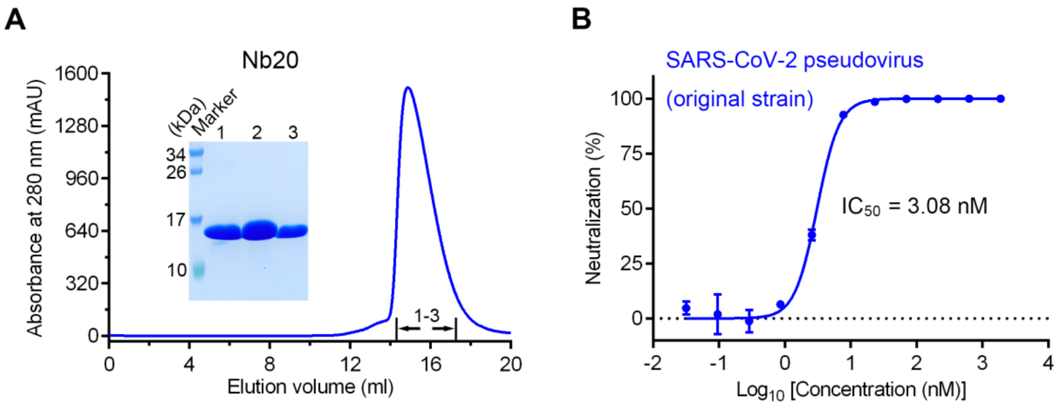

Supplementary Figure 6. Solution behavior and pseudovirus neutralization of Nb20. **(A)** Purification and gel-fitration characterization of Nb20. The inset figure shows the SDS-PAGE analyses of the pooled samples. **(B)** Neutralization of SARS-CoV-2 pseudovirus (original strain) by Nb20. The error bar stands for the mean ± SD from triplicate experiences.

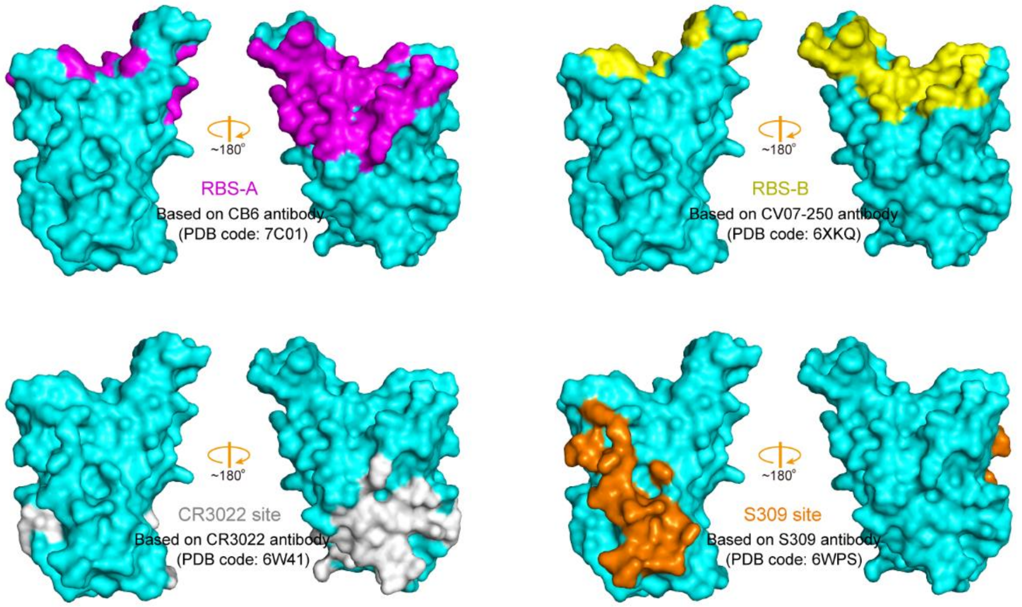

Supplementary Figure 7. The currently-defined antigenic sites on S-RBD of SARS-CoV-2. For easy comparison, the S-RBD molecules are oriented similarly and rendered as molecular surface in cyan. The antigenic sites are depicted as follows: RBS-A epitope in magenta, RBS-B epitope in yellow, CR3022 site in gray, and S309 site in orange. The epitope of RBS-C is shown in main-text figure 4.

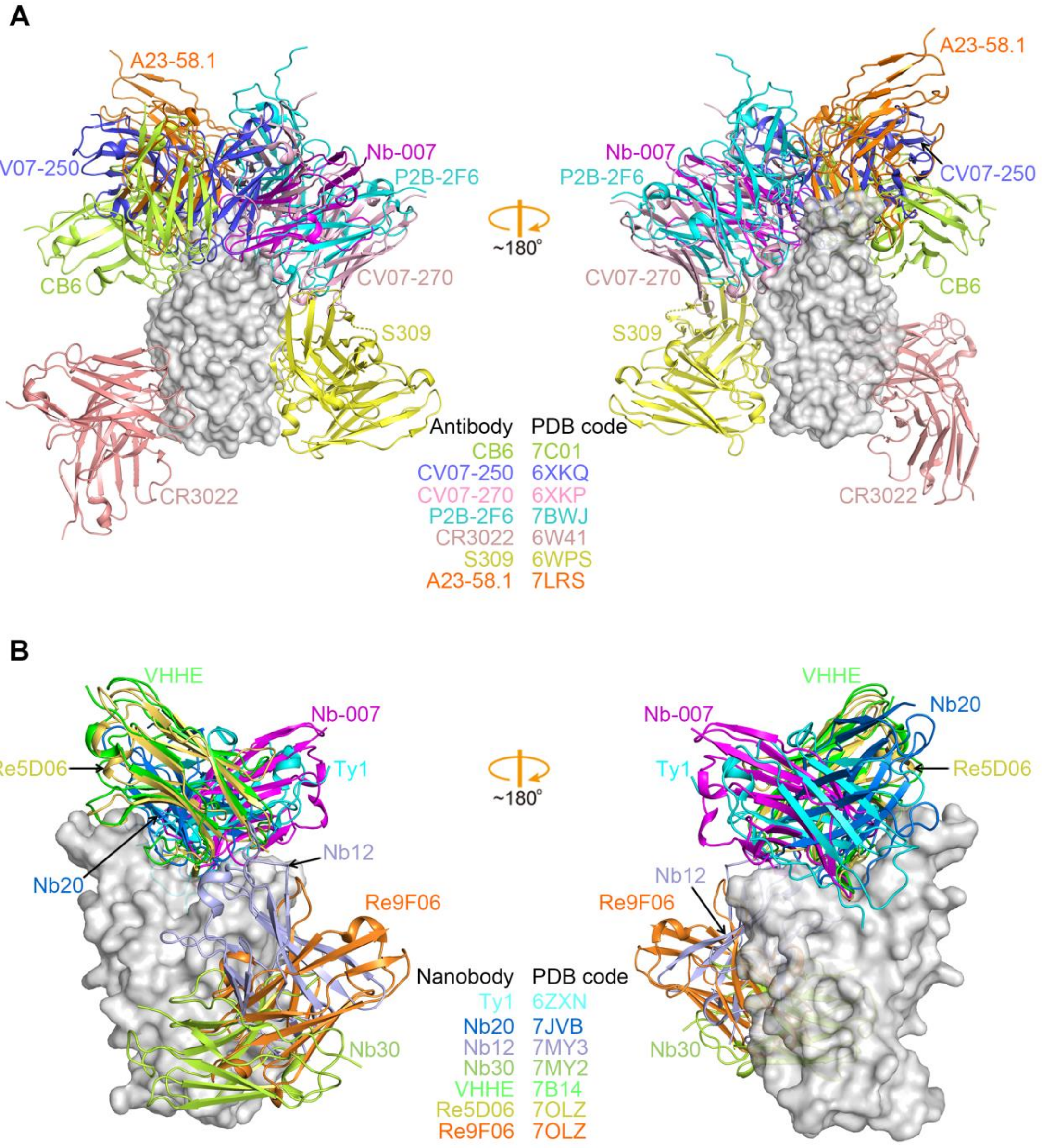

Supplementary Figure 8. Comparison of the epitope of Nb-007 with those of the indicated antibodies and nanobodies. **(A)** Superimposition of the structures of S-RBD (gray) in complex with Nb-007 (magenta) and antibodies CB6 (lemon, PDB code: 7C01), CV07-250 (blue, PDB code: 6XKQ), CV07-270 (light-pink, PDB code: 6XKP), P2B-2F6 (cyan, PDB code: 7BWJ), CR3022 (salmon, PDB code: 6W41), S309 (yellow, PDB code: 6WPS) and A23-58.1 (orange, PDB code: 7LRS). **(B)** Superimposition of the structures of S-RBD (gray) in complex with nanobodies Ty1 (cyan, PDB code: 6ZXN), Nb20 (blue, PDB code: 7JVB), Nb12 (light-blue, PDB

code: 7MY3), Nb30 (lemon, PDB code: 7MY2), VHHE (green, PDB code: 7B14), Re5D06 (yellow, PDB code: 7OLZ), Re9F06 (orange, PDB code: 7OLZ), and Nb-007 (magenta). Antibodies and nanobodies are shown in cartoon and RBD is shown in surface representation.

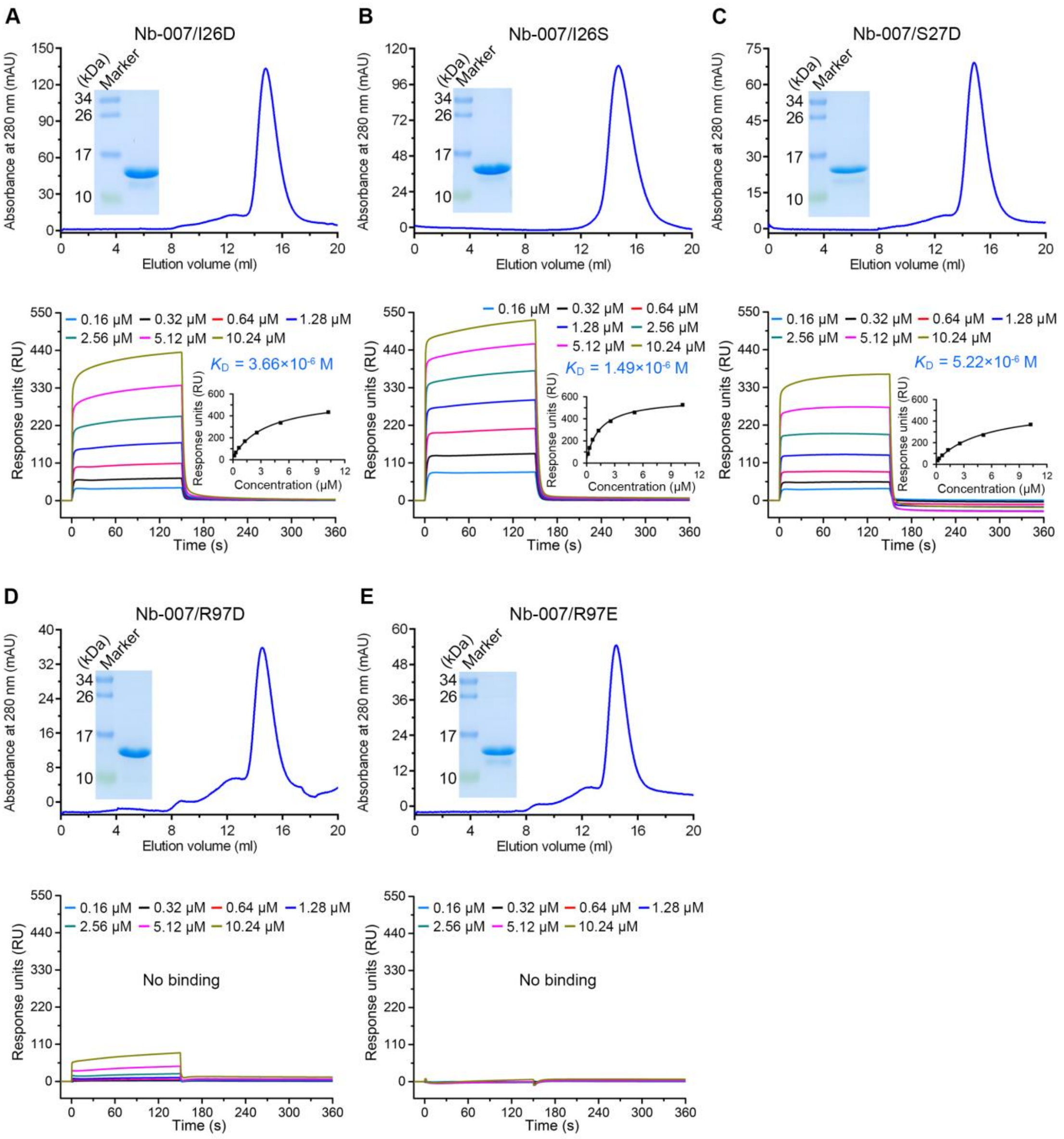

Supplementary Figure 9. Solution behaviors and binding capacity of Nb-007 mutants. **(A-E)** Characterization of the solution behavior of Nb-007/I26D (A), Nb-007/I26S (B), Nb-007/S27D (C), Nb-007/R97D (D), Nb-007/R97E (E) by gel filtration chromatography (upper panel) and the affinity calculation of the binding of individual mutants to SARS-CoV-2 Beta variant S-RBD using SPR (lower panel).

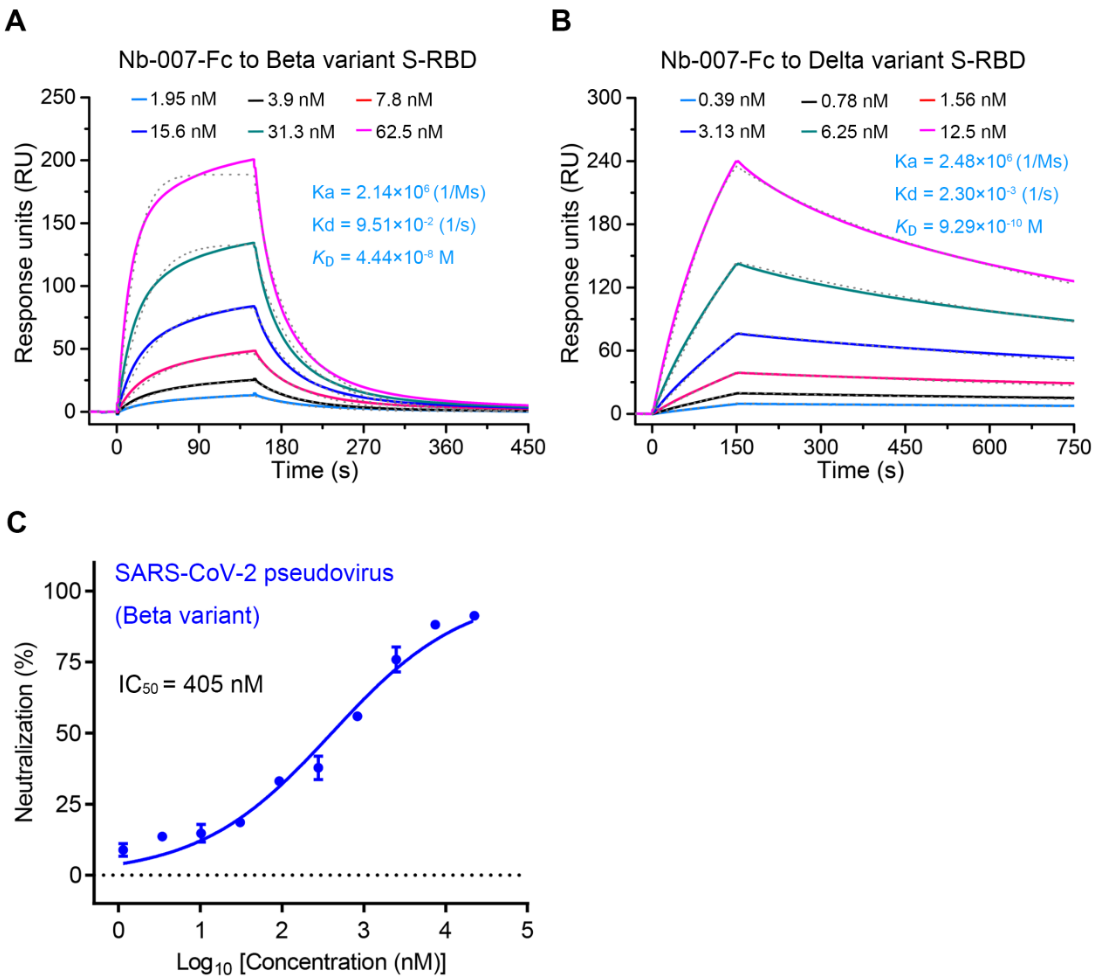

Supplementary Figure 10. Binding capacity and neutralizing activity of Nb-007-Fc against SARS-CoV-2 variants. **(A, B)** Affinity analysis of the binding of Nb-007-Fc to SARS-CoV-2 Beta variant S-RBD (A) and Delta variant S-RBD (B) using SPR. The real-time binding kinetics are shown. **(C)** Inhibition of the pseudovirus entry by Nb-007-Fc at the indicated concentrations for the SARS-CoV-2 Beta variant. Error bar represents the mean ± SD of triplicate.

Supplementary Table 1. Data collection and structure refinement statistics.

	SARS-CoV-2 S-RBD with Nb-007 (PDB code: 7W1S)
**Data collection**	
Space group	$P4_12_12$
Cell dimensions	
a, b, c (Å)	56.80, 56.80, 219.77
α, β, γ (°)	90.00, 90.00, 90.00
Wavelength (Å)	0.97915
Resolution (Å)	50.00-2.00 (2.07-2.00)
Unique reflections	25547 (2451)
R_{merge}	0.182 (0.739)
$CC_{1/2}$	0.999 (0.900)
I/sig$I$	16.50 (2.00)
Completeness (%)	99.9 (99.2)
Redundancy	17.4 (11.4)
**Refinement**	
Resolution (Å)	29.65-2.00
No. reflections	25292
R_{work}/R_{free}	0.199/0.223
No. atoms	
Protein	2388
Ligand/ion	0
Water	268
B-factors	
Protein	28.5
Ligand/ion	-
Water	39.1
R.m.s. deviations	
Bond lengths (Å)	0.004
Bond angles (°)	0.567
Ramachandran plot (%)	
Favored region	98.01
Allowed region	1.99
Outlier region	0

A single crystal was used to collect the data.

Values in parentheses are for the highest-resolution shell.
